# A phase II study of FOLFOXIRI plus bevacizumab as initial chemotherapy for patients with untreated metastatic colorectal cancer: TRICC1414 (BeTRI)

**DOI:** 10.1007/s10147-020-01811-w

**Published:** 2020-10-23

**Authors:** Katsunori Shinozaki, Takeshi Yamada, Junichiro Nasu, Toshihiko Matsumoto, Yasuhiro Yuasa, Takeshi Shiraishi, Hiroaki Nagano, Ichiro Moriyama, Toshiyoshi Fujiwara, Masashi Miguchi, Ryosuke Yoshida, Kimiyasu Nozaka, Hiroaki Tanioka, Takeshi Nagasaka, Yasuro Kurisu, Michiya Kobayashi, Kenji Tsuchihashi, Michio Inukai, Takashi Kikuchi, Tomohiro Nishina

**Affiliations:** 1 Division of Clinical Oncology, Hiroshima Prefectural Hospital, 1-5-54 Ujinakanda, Hiroshima, 734-8530 Japan; 2 Division of Gastroenterology, University of Tsukuba, Tsukuba, 305-8577 Japan; 3 Department of Internal Medicine, Okayama Saiseikai General Hospital, Okayama, 700-8501 Japan; 4 Department of Internal Medicine, Himeji Red Cross Hospital, Himeji, 670-8540 Japan; 5 Department of Surgery, Tokushima Red Cross Hospital, Tokushima, 773-8502 Japan; 6 Department of Medical Oncology, Matsuyama Red Cross Hospital, Matsuyama, 790-8520 Japan; 7 Department of Gastroenterological Surgery, Yamaguchi University Graduate School of Medicine, Ube, 755-8505 Japan; 8 Innovative Cancer Center, Shimane University Hospital, Izumo, 693-0021 Japan; 9 Department of Gastroenterological Surgery, Okayama University Graduate School of Medicine, Dentistry and Pharmaceutical Sciences, Okayama, 700-8530 Japan; 10 Department of Surgery, Hiroshima City Asa Citizens Hospital, Hiroshima, 731-0293 Japan; 11 Department of Surgery, Okayama Rosai Hospital, Okayama, 702-8055 Japan; 12 Department of Surgery, Sanin Rosai Hospital, Yonago, 683-8605 Japan; 13 Department of Clinical Oncology, Kawasaki Medical School Hospital, Kurashiki, 701-0192 Japan; 14 Department of Surgery, Hamada Medical Center, Hamada, 697-8511 Japan; 15 Cancer Treatment Center, Kochi Medical School Hospital, Nankoku, 783‑8505 Japan; 16 Department of Hematology, Oncology and Cardiovascular Medicine, Kyushu University Hospital, Fukuoka, 812-8582 Japan; 17 Foundation for Biomedical Research and Innovation at Kobe for Medical Innovation, Kobe, 650-0047 Japan; 18 Department of Gastrointestinal Medical Oncology, National Hospital Organization Shikoku Cancer Center, Matsuyama, 791-0280 Japan

**Keywords:** FOLFOXIRI plus bevacizumab, Metastatic colorectal cancer, Efficacy, Conversion surgery, Safety

## Abstract

**Purpose:**

FOLFOXIRI plus bevacizumab is regarded as a first-line therapeutic option for selected patients with metastatic colorectal cancer (mCRC). Our aim was to assess the efficacy and safety of induction treatment with FOLFOXIRI plus bevacizumab in patients with untreated mCRC harboring *UGT*1*A*1 wild (*1/*1), or single-hetero (*1/*6 or *1/*28) genotypes.

**Methods:**

Twelve cycles of FOLFOXIRI plus bevacizumab were administered to patients with untreated mCRC. The primary endpoint was the overall response rate (ORR) assessed by central independent reviewers. Secondary endpoints included time to treatment failure (TTF), progression-free survival (PFS), overall survival (OS), relative dose intensity (RDI), R0 resection rate, and safety. The exploratory objectives were early tumor shrinkage (ETS) and depth of response (DoR).

**Results:**

Of the 47 patients enrolled, 46 and 44 patients were eligible for the safety and efficacy analysis, respectively. The primary endpoint was met. The ORR was 63.6% (95% CI 47.8–77.6). At a median follow-up of 25.4 months, median TTF, PFS, and OS was 8.1, 15.5, and 34.4 months, respectively. The median RDI of 5-fluorouracil, irinotecan, oxaliplatin, and bevacizumab was 72, 69, 62, and 71%, respectively. R0 resection rate was 22.7%. Grade 3 or higher adverse events (≥ 10%) included neutropenia (65.2%), febrile neutropenia (26.1%), leukopenia (23.9%), anorexia (10.9%), nausea (10.9%), and diarrhoea (10.9%). No treatment-related deaths were observed. ETS and DoR were 70.5 and 45.4%, respectively.

**Conclusions:**

FOLFOXIRI plus bevacizumab induction treatment of Japanese patients was shown to be beneficial and manageable, although caution is required since the treatment causes febrile neutropenia.

**Electronic supplementary material:**

The online version of this article (10.1007/s10147-020-01811-w) contains supplementary material, which is available to authorized users.

## Introduction

Colorectal cancer (CRC) is the third most common malignant neoplasm and the second major cause of cancer death in 2018 worldwide for both sexes [1]. Most patients with metastatic CRC (mCRC) have unresectable disease and cannot be cured. However, long-term survival or even cure is reported to have been attained in 20–50% of patients who underwent complete R0 resection of their metastases [2].

The Gruppo Oncologico Nord Ovest (GONO) group’s phase III TRIBE trial compared FOLFOXIRI plus bevacizumab versus FOLFIRI plus bevacizumab in unresectable mCRC patients in Italy [3]. This trial demonstrated that FOLFOXIRI plus bevacizumab increased ORR (65 vs. 53%, *p* = 0.006) and PFS (12.1 vs. 9.7 months, *p* = 0.003). An updated analysis on the TRIBE trial reported that OS was prolonged (29.8 vs. 25.8 months, *p* = 0.03) [4].

The major clinical practice guidelines, including the Japanese Society for Cancer of the Colon and Rectum (JSCCR) guideline 2019, all recommend FOLFOXIRI plus bevacizumab as a first-line therapeutic option for selected patients with mCRC [5–8]. Only two prospective, single-arm phase II trials of FOLFOXIRI plus bevacizumab have been conducted to assess the safety and efficacy in Japan. One is the QUATTRO study using the GONO-FOLFOXIRI regimen [9]. The other is the JACCRO-CC11 trial using a modified FOLFOXIRI regimen that consisted of oxaliplatin (85 mg/m^2^), irinotecan (150 mg/m^2^), l-leucovorin (200 mg/m^2^), and 5-fluorouracil (2400 mg/m^2^) [10]. Both studies concluded that FOLFOXIRI plus bevacizumab was effective. The incidence of febrile neutropenia was as low as 5% in modified FOLFOXIRI compared to 21.7% in GONO-FOLFOXIRI. However, mature PFS and OS have not been documented for either of these studies. The recommended doses of FOLFOXIRI also remain controversial.

Adverse events associated with the FOLFOXIRI regimen include higher risks of neutropenia and diarrhoea [11]. Irinotecan especially has significant adverse effects, including myelosuppression and diarrhoea. In Japanese patients, homozygosity for *UGT*1*A*1*28 or *UGT*1*A*1*6 and heterozygosity for both *UGT*1*A*1*6 and *UGT*1*A*1*28 are associated with severe irinotecan-related neutropenia [12, 13].

Thus, we conducted a single-arm, multicenter, phase II study to assess the efficacy and safety of induction treatment with FOLFOXIRI plus bevacizumab in patients with untreated mCRC harboring *UGT*1*A*1 wild (*1/*1) or single-hetero (*1/*6 or *1/*28) genotypes. In our study, sequential treatment with the remaining drugs was continued after termination of protocol therapy or discontinuation of oxaliplatin and/or irinotecan at the investigator’s discretion.

## Patients and methods

### Study design

The Bevacizumab plus Triplet (BeTRI) study was a multi-site, open-label, single-arm, phase II clinical trial. This trial was registered on the UMIN Clinical Trials Registry (UMIN000017102), followed by Japan Registry of Clinical Trials (jRCTs061180021).

### Patients

Patients who met the following criteria were enrolled in this study: aged between 20 and 70 years; histologically confirmed adenocarcinoma of the colon or rectum; unresectable or recurrent CRC patient; no prior chemotherapy, immunotherapy, or radiation therapy (but can be enrolled 6 months after the date of completion of adjuvant chemotherapy); one or more measurable lesions; ECOG PS of 0 or 1; patients harbored *UGT*1*A*1 wild (*1/*1) or single-heterozygous (*1/*6 or *1/*28) genotypes. Patients were ineligible if they had severe, uncontrolled organ or metabolic dysfunction.

### Endpoints

The primary endpoint was the objective response rate (ORR) assessed by central independent reviewers. Secondary endpoints were TTF and PFS, OS, R0 resection rate, relative dose intensity (RDI), and safety. TTF represented the time from the initial day of protocol therapy to the first day when we observed any of the following events: discontinuation of protocol therapy; initial progression; death with any cause. PFS was defined as the time from the initial day of protocol therapy to the initial progression day or death for any reason. The exploratory objectives were early tumor shrinkage (ETS) and depth of response (DoR).

### Treatment and evaluation

We defined protocol therapy as 12 cycles of FOLFOXIRI plus bevacizumab induction treatment consisting of a 30–90-min infusion of bevacizumab at a dose of 5 mg/kg, a 90-min infusion of irinotecan at a dose of 165 mg/m^2^, a 120-min infusion of oxaliplatin at a dose of 85 mg/m^2^ and a concomitant 120-min infusion of *l*-leucovorin at a dose of 200 mg/m^2^. These were followed by a 48-h continuous infusion of 5-fluorouracil to a total dose of 3200 mg/m^2^. Cycles were repeated every 14 days.

The chemotherapy was continued until disease progression, an unacceptable adverse event, tumor resection, a delay of more than 29 days due to an adverse event, reduction of 5-fluorouracil dose to less than 2000 mg/m^2^, or consent withdrawal. In cases of prespecified adverse events, treatment modification was permitted according to the study protocols (Supplementary Tables 1 and 2). If oxaliplatin and/or irinotecan was discontinued at the investigator's discretion, sequential treatment consisting of the remaining drugs was continued.

We analyzed safety with a safety analysis set (SAS) in which patients received at least one cycle of protocol therapy. Efficacy was analyzed with a full analysis set (FAS) consisting of eligible patients. Based on the Response Evaluation Criteria in Solid Tumors (RECIST) version 1.1, central independent reviewers assessed response and progression, referring to CT or MRI taken every 8 weeks. ETS was defined as a reduction of at least 20% in the sum of the longest diameters of target lesions at week 8 compared with baseline. DoR was defined as the relative change in the sum of the longest diameters of RECIST target lesions at the nadir, in the absence of new lesions or progression of non-target lesions, when compared with baseline. Adverse events were evaluated according to CTCAE version 4.0.

### Sample size

We assumed the expected response rate to be 70% and the threshold response rate to be 45%, with a two-sided α error of 0.05 and a power of 90%. The expected response rate was grounded in the results of the GONO and TRIBE trials, of which the confirmed ORRs were 65 and 77% for FOLFOXIRI plus bevacizumab, respectively [3, 14]. We also referred to the threshold response rate reported by the NO16966 and TRIBE trials, of which the confirmed ORRs were 34 and 53% for doublet plus bevacizumab, respectively [3, 15]. We estimated a minimum of 40 patients, but we planned to enrol 45 or more patients, taking account of dropouts and withdrawals.

### Statistical analyses

We concluded that the treatment could be regarded as promising if the lower limit of the 95% CI exceeded 45%. For the evaluation of the ORR and ETS, we calculated the 95% CI of the rate using the Clopper–Pearson method with F distribution. We used the Kaplan–Meier method to estimate TTF, PFS, and OS. Statistical analyses were carried out using SAS version 9.4 (SAS Institute Inc., Cary, NC, USA) and R × 64 version 3.5.2.

## Results

### Patients

Between April 2015 and May 2017, a total of 47 patients were enrolled from 16 Japanese study sites. One patient was excluded from the study because of a deviation of the inclusion criteria. Two more patients were removed after the central reviewers determined there were no measurable lesions. The numbers in the SAS and FAS were, therefore, 46 and 44, respectively (Supplementary Fig. 1).

Table 1 shows patient characteristics in the SAS. There were twenty-six male and twenty female patients with a median age of 58 years (range 29–68 years). ECOG PS scores of 0 and 1 were 76% and 24%, respectively. The site of the primary tumor was right colon in 10 (22%) patients, left colon in 16 (35%), and rectum in 20 (43%) patients. Resection rate of the primary tumor was 37%. Seventeen (37%), 24 (52%) and 5 (11%) patients had *RAS* wild-type, *RAS* mutated, and *RAS* unknown tumor, respectively.

**Table 1:** Baseline characteristics

	Patients (*n* = 46)	%
Age (years; median, range)	58 (29–68)	
Sex		
Male	26	57
Female	20	43
ECOG PS		
0	35	76
1	11	24
Site of primary tumor		
Right colon	10	22
Left colon	16	35
Rectum	20	43
Resection of primary tumor		
Yes	17	37
No	29	63
Site of metastases*		
Liver	36	78
Lung	13	28
Lymph node	7	15
Peritoneum	4	9
*UGT*1*A*1		
Wild type (*1/*1)	24	52
Single heterozygous (*1/*6 or *1/*2*8*)	22	48
*RAS*		
Wild type	17	37
Mutated	24	52
Unknown	5	11

*ECOG PS* Eastern Cooperative Oncology Group performance status, *Right colon* cecum, ascending and transverse colon, *Left colon* descending and sigmoid colon

*Site of metastases were counted in duplicate

### Study treatment

All 46 enrolled patients received FOLFOXIRI plus bevacizumab. The commencement of second cycle treatment was delayed in 33 (72%) patients. In addition, the dose level was reduced in the second cycle in 23 (50%) patients. Meanwhile, twelve (26%) patients completed the second cycle treatment as scheduled without dose modification.

A total of 27 (59%) patients completed twelve cycles of protocol therapy. Table 2 shows the details of the treatment after protocol therapy. At the twelfth cycle, 18 (39.1%) patients received FOLFOXIRI plus bevacizumab, six (13.0%) patients received FOLFIRI plus bevacizumab, two (4.3%) patients received FOLFIRI, and one (2.2%) patient received infusional 5-fluorouracil and leucovorin plus bevacizumab. The protocol therapy was discontinued in 9 (20%) patients due to adverse events, in 8 (17%) patients due to surgery, in one (2%) patient due to progressive disease, and in one (2%) patient due to withdrawal of consent. Of the nine patients for whom protocol therapy was discontinued due to adverse events, eight patients resumed chemotherapy, and their treatments (number of patients) were as follows: FOLFOXIRI plus bevacizumab (3); FOLFOXIRI (1); FOLFIRI plus bevacizumab (2); Capecitabine plus bevacizumab (1); and 5-fluorouracil and leucovorin plus bevacizumab (1).

**Table 2:** Reasons of protocol discontinuation and post-protocol treatment details

Reason of protocol discontinuation	*n* = 46	Post-treatment details	*n* = 46	%
Surgery	8	R0 resection	5	10.9
		R2 resection	3	6.5
Patient's withdrawal	1	FOLFIRI + Bmab	1	2.2
Disease progression	1	FOLFOXIRI + Bmab	1	2.2
Adverse event	9	FOLFOXIRI ± Bmab	4	8.7
		FOLFIRI + Bmab	2	4.3
		FL + Bmab	1	2.2
		Capecitabine + Bmab	1	2.2
		BSC	1	2.2
Protocol completion*	27	FOLFOXIRI + Bmab	18	39.1
		FOLFIRI ± Bmab	8	17.4
		FL + Bmab	1	2.2

*Treatment performed in the 12th cycle of protocol therapy

*R0 resection* curative resection, *Bmab* bevacizumab, *FL* infusional 5-fluorouracil and leucovorin, *BSC* best supportive care

Although second-line treatments were not specified by the protocol, we requested that treatment administered after progression of protocol therapy and treatment with drugs not included in the protocol therapy (i.e. second-line treatments) must be reported. Second-line chemotherapy (cases per 16 patients total) were as follows: FOLFIRI plus anti-VEGF/VEGFR (4); FOLFOXIRI plus or minus bevacizumab (3); anti-EGFR plus or minus irinotecan (3); capecitabine plus or minus bevacizumab (3); irinotecan plus tegafur/gimeracil/oteracil (1); regorafenib (1); and trifluridine/tipiracil (1) (Supplementary Table 4).

In the FAS, the median relative dose intensity of 5-fluorouracil, irinotecan, oxaliplatin, and bevacizumab was 72, 69, 62 and 71%, respectively. The median number of cycles administered per patient as FOLFOXIRI plus or minus bevacizumab was eight (range 2–12).

### Efficacy

**Fig. 1:**
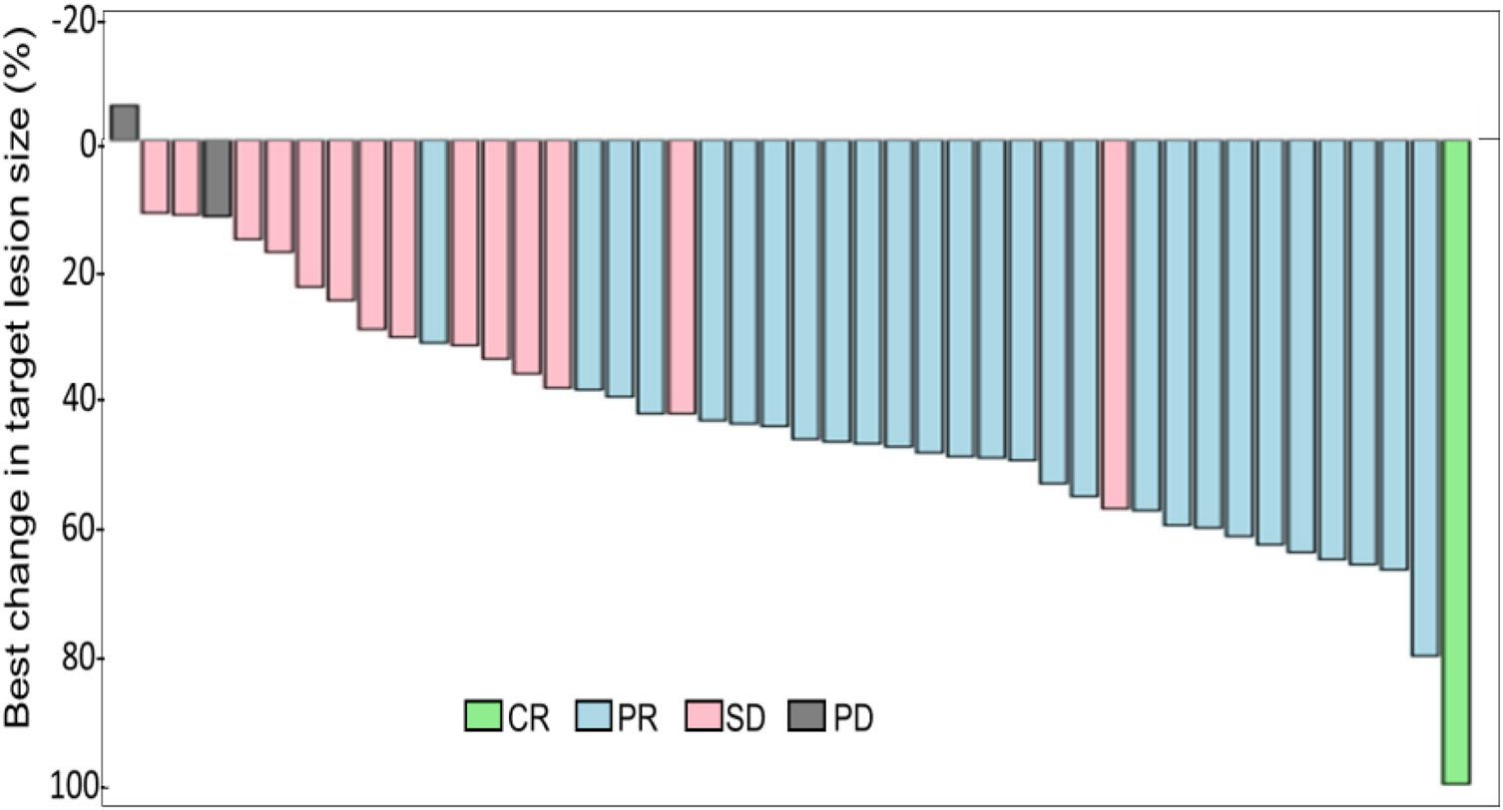
Waterfall plot showing maximum percentage change from baseline in size of tumors assessed by central independent reviewers

The efficacy results are summarized in Table 3. Because CR and PR were achieved in 1 (2.3%) patient and 27 (61.4%) patients, respectively, the ORR was 63.6% (95% CI 47.8–77.6). The numbers (%) of patients with stable disease (SD) and with progressive disease (PD) were 14 (31.8%) and 2 (4.5%), respectively. In addition, the ORR at week 8 was 54.5%. ETS was 70.5% (95% CI 54.8–83.2). A waterfall plot of maximum tumor reduction rate is presented in Fig. 1. Median DoR was 45.4% (95% CI 37.8–49.9).

**Table 3:** Tumor response (ORR, ETS, DoR), TTF, PFS, and OS

Variable	All (*n* = 44)	Location		*RAS* status*	
		Right-sided colon (*n* = 10)	Left-sided colon (*n* = 34)	Wild (*n* = 15)	Mutated (*n* = 24)
Overall response rate (ORR)					
No. of pts (%) (95% CI)	28 (63.6) (47.8–77.6)	7 (70.0) (34.8–93.3)	21 (61.8) (43.6–77.8)	11 (73.3) (44.9–92.2)	15 (62.5) (40.6–81.2)
Early tumor shrinkage (ETS)					
No. of pts (%) (95% CI)	31 (70.5) (54.8–83.2)	7 (70.0) (34.8–93.3)	24 (70.6) (52.5–84.9)	11 (73.3) (44.9–92.2)	17 (70.8) (48.9–87.4)
Deepness of response (DoR)					
Median % (95% CI)	45.4 (37.8–49.9)	44.7 (31.1–64.7)	45.4 (36.1–49.3)	47.6 (39.3–61.1)	45.2 (34.1–50.8)
Time to treatment failure (TTF)					
Median months (95% CI)	8.1 (5.3–10.1)	11.5 (8.2–24.1)	6.4 (4.2–9.7)	11.4 (7.6–15.5)	6.8 (4.2–9.3)
Progression-free survival (PFS)					
Median months (95% CI)	15.5 (11.5–23.4)	24.1 (11.5–26.2)	14.9 (10.1–22.9)	13.2 (11.4–24.1)	17.9 (13.7–26.2)
Overall survival (OS)					
Median months (95% CI)	34.4 (26.4-NR)	26.4 (26.2-NR)	34.4 (23.6-NR)	37.1 (23.6-NR)	29.8 (26.2-NR)

*Right-sided colon* cecum, ascending and transverse colon, *Left-sided colon* descending, sigmoid colon, and rectum, *No*. number, *pts* patients, *NR* not reached

*Five patients with unknown *RAS* status were excluded

**Fig. 2:**
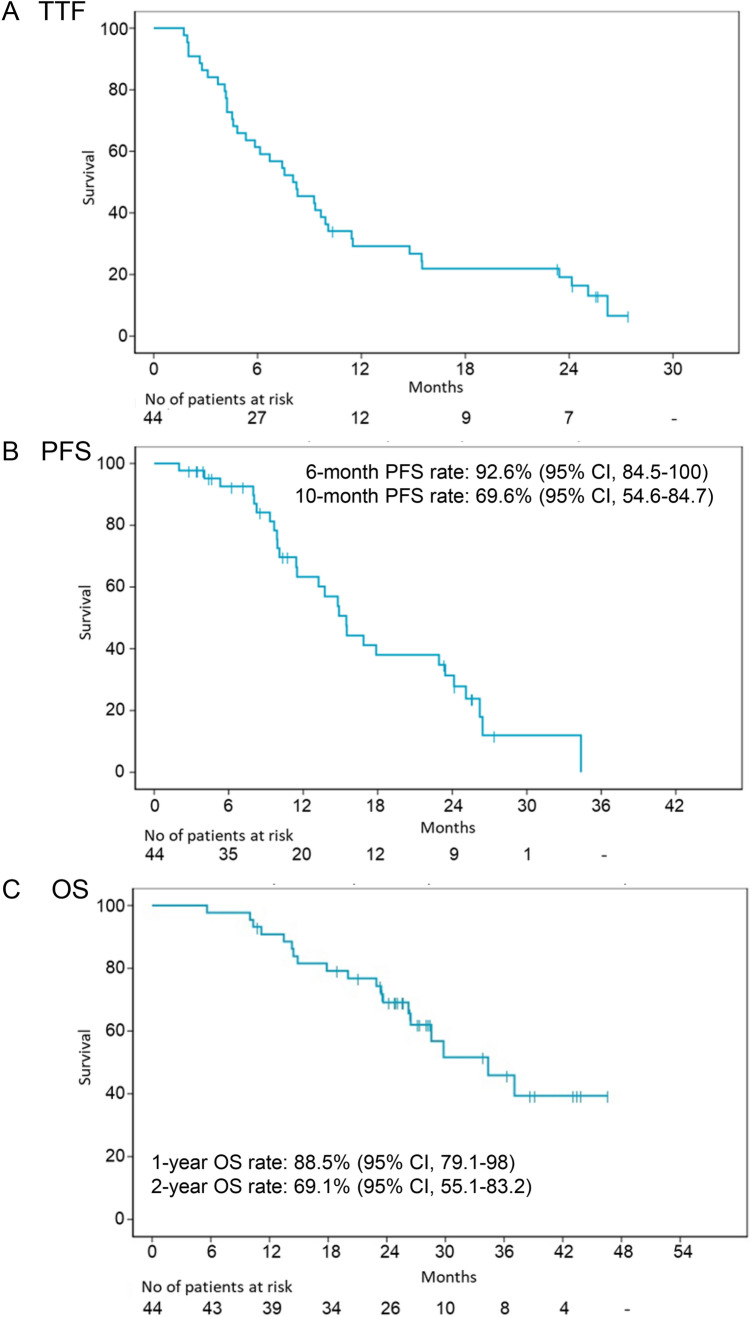
Kaplan–Meier estimates of time to treatment failure (**a**), progression-free survival (**b**), and overall survival (**c**)

The median follow-up period was 25.4 months (ranging from 6 to 47 months). Median TTF was 8.1 months (95% CI 5.3–10.1) based on 38 events (86.4%) among 44 patients (Fig. 2a). Median PFS was 15.5 months (95% CI 11.5–23.4) based on 28 events (63.6%) among 44 patients (Fig. 2b). Median OS was 34.4 months (95% CI 26.4 not reached) based on 19 deaths among 44 patients (43.2%) (Fig. 2c).

## Subgroup analyses by primary tumor sidedness and *RAS* mutational status

We evaluated the activity of FOLFOXIRI plus bevacizumab in an ad hoc analysis on the basis of primary tumor sidedness or *RAS* status (Table 3). Following classification of the 44 patients in the FAS on the basis of primary tumor sidedness and *RAS* mutation status, left-sided colon tumors were associated with a higher frequency of mutated *RAS* tumors than right-sided colon tumors (47.7 vs. 6.8%) (Supplementary Table 3). In stratification by sidedness, the ORR was numerically higher in right-sided colon tumors compared with left-sided ones (70.0 vs. 61.8%, respectively). As for ETS and DoR, there was no difference between the two sides.

In stratification by *RAS* status, the ORR was numerically higher in *RAS* wild-type tumors compared with *RAS* mutated ones (73.3 vs. 62.5%). ETS and median DoR were 73.3 and 47.6% in *RAS* wild-type tumors and 70.8 and 45.2% in *RAS* mutated tumors, respectively.

When analyzed on the basis of primary tumor sidedness, median PFS was 24.1 months (95% CI 11.5–26.2) and 14.9 months (95% CI 10.1–22.9) in right- and left-sided colon tumor cases, respectively, and the two survival curves intersected in the follow-up period (Supplementary Fig. 2a). Median OS was 26.4 months (95% CI 26.2 not reached) and 34.4 months (95% CI 23.6 not reached) in right- and left-sided colon tumor cases, respectively (Supplementary Fig. 2c). When analyzed according to *RAS* mutational status, median PFS was 13.2 months (95% CI 11.4–24.1) and 17.9 months (95% CI 13.7–26.2) in *RAS* wild-type and mutated tumor cases, respectively (Supplementary Fig. 2b), and the two survival curves crossed. Median OS was 37.1 months (95% CI 23.6 not reached) and 29.8 months (95% CI 26.2 not reached) in *RAS* wild-type and mutated tumor cases, respectively (Supplementary Fig. 2d).

### Conversion surgery

Fourteen patients (31.8%) had conversion surgery performed with curative intent. Nine of these patients underwent surgery during the protocol therapy, and two patients did after the end of protocol therapy. The remaining three patients did during sequential treatment following the completion of protocol therapy. The median time to surgery from the initial day of protocol therapy was 182.5 days (range 87–316 days).

R0 resection was performed in 10 (22.7%) patients. Five of these patients were treated with 12 cycles of protocol therapy (two patients) or sequential treatment (three patients). When we examined the presence of ETS in ten patients with R0 resection, eight patients had ETS and two patients had no ETS. The surgical procedures (number of patients) performed on the four patients who could not achieve R0 resection included primary resection (3) and liver and lung resection (1).

## Toxicity

All observed adverse events in the SAS are summarized in Table 4. Treatment-related grade 3 or 4 adverse events occurring in at least 10% of patients were as follows: neutropenia (65.2%); febrile neutropenia (26.1%); leukopenia (23.9%); nausea (10.9%); anorexia (10.9%); and diarrhea (10.9%). Two (16.7%) of the 12 patients who developed febrile neutropenia were treated with G-CSF. No new adverse events were observed compared with previous historical studies.

**Table 4:** Adverse events (≥ 5%) occurring in the safety sets (*n* = 46)

Adverse event	All grade *n* (%)	≥ G3 *n* (%)
Anemia	45 (97.8)	2 (4.3)
Neutropenia	42 (91.3)	30 (65.2)
Leukopenia	35 (76.1)	11 (23.9)
Thrombocytopenia	26 (56.5)	0
Peripheral sensory neuropathy	39 (84.8)	1 (2.2)
Malaise	38 (82.6)	0
Alopecia	36 (78.3)	0
Nausea	35 (76.1)	5 (10.9)
Anorexia	35 (76.1)	5 (10.9)
Diarrhea	27 (58.7)	5 (10.9)
Fatigue	27 (58.7)	2 (4.3)
Mucositis oral	25 (54.3)	2 (4.3)
Hypertension	25 (54.3)	4 (8.7)
Vomiting	14 (30.4)	2 (4.3)
Febrile neutropenia	12 (26.1)	12 (26.1)
Constipation	9 (19.6)	0
Fever	9 (19.6)	2 (4.3)
Hiccups	8 (17.4)	0
Epistaxis	7 (15.2)	0
Palmar–plantar erythrodysesthesia syndrome	7 (15.2)	0
Skin hyperpigmentation	5 (10.9)	0
Thromboembolic event	5 (10.9)	2 (4.3)
Dry skin	4 (8.7)	0
Hypoalbuminemia	3 (6.5)	0
Abdominal pain	3 (6.5)	0
Dysgeusia	3 (6.5)	0

In total, 14 serious adverse events occurred in 11 patients (23.9%). Five of these adverse events were independent of protocol therapy. The remaining adverse events included infections (4), a thromboembolic event (1), an anastomosis fistula (1), ileus (1), nausea (1), and fever (1). All patients recovered from the serious adverse events at the end of the study. No treatment-related deaths occurred in the present study.

## Discussion

Our BeTRI study confirmed the efficacy and safety of GONO-FOLFOXIRI plus bevacizumab in Japanese mCRC patients harboring *UGT*1*A*1 wild (*1/*1) or single-heterozygous (*1/*6 or *1/*28) genotypes.

Right-sided colon tumors are associated with a poorer prognosis compared with left-sided tumors [16, 17]. Considering only patients with previously untreated mCRC receiving first-line doublet chemotherapy plus or minus bevacizumab, Loupakis et al. observed that ORR and PFS were statistically significantly higher in patients with left-sided tumors [16]. Interestingly, our ad hoc analysis showed that FOLFOXIRI plus bevacizumab treatment produced a numerically higher ORR, TTF, and PFS in right-sided colon tumor cases (compared with left-sided tumors). In right-sided colon tumor cases, ORR was the same, irrespective of *RAS* mutational status. This finding supports the post hoc analysis of the TRIBE trial which found that FOLFOXIRI plus bevacizumab may be able to efficiently counteract the intrinsic aggressiveness of right-sided colon tumors [18].

FOLFOXIRI plus bevacizumab with an expected ORR of around 70% is an attractive strategy for conversion chemotherapy. Interestingly, conversion surgery (31.8%) and R0 resection (22.7%) rate in the present study were consistent with previous studies [9, 10, 14], despite the decreased dose intensity. The median time to surgery from the initial day of protocol therapy was 182.5 days (range 87–316 days). Of the 14 patients who underwent surgery, the chemotherapy regimen immediately prior to surgery was FOLFOXIRI (*n* = 11), FOLFIRI (*n* = 1), and 5-fluorouracil and leucovorin (*n* = 2). Among the 10 patients who had undergone R0 resection, 50% of patients were treated with 12 or more cycles of protocol therapy. The ten patients who underwent R0 resection included two patients without ETS. This finding supports previously published meta-regression analysis data suggesting that a high number of chemotherapy cycles (close to 12) is associated with conversion surgery [19]. Therefore, a sufficient treatment time period may be required for R0 resection in unresectable or recurrent CRC.

Considering the safety profile of this drug regimen, 26.1% of Japanese patients developed febrile neutropenia in our BeTRI study, which was consistent with the results of the QUATTRO study. It should be noted that only two (16.7%) of 12 patients were treated with G-CSF as a primary treatment or secondary prophylaxis of febrile neutropenia. The second cycle of treatment was performed without modification of dose or delay in 12 (26%) patients. The median relative dose intensities of 5-fluorouracil, irinotecan, oxaliplatin, and bevacizumab were 72, 69, 62, and 71%, respectively. Compared with earlier studies [9, 14], the frequency of G-CSF use was extremely low. This may be related to the lower dose intensities in comparison with previous studies. The observed incidence of grade 3 or more diarrhea was low (10.9%) compared with the two GONO-FOLFOXIRI plus bevacizumab studies (14–18.8%) [4, 14], and was comparable with the QUATTRO study [9]. The major differences from the TRIBE trial was that target patients who harbored *UGT*1*A*1*1/*1, *1/*6, or *1/*28 were included in our BeTRI and QUATTRO studies. This inclusion criterion may be the reason for the lower frequency of diarrhea. Our results indicate that the GONO-FOLFOXIRI regimen could be managed with appropriate dose reductions and treatment delays in Japanese patients harboring *UGT*1*A*1 wild (*1/*1), or single-hetero (*1/*6 or *1/*28) genotypes.

In clinical trials, chemotherapy is historically administered until disease progression, unacceptable toxicities, or patients’ refusal. Oxaliplatin often causes cumulative neurotoxicity before clinical progression [20]. NCCN and ASCO guidelines recommend discontinuation of oxaliplatin from FOLFOX or CAPEOX three–four months after initiation of treatment or sooner for unacceptable neurotoxicity, with other drugs in the regimen maintained until time of tumor progression [6, 7]. In contrast, continuation of FOLFIRI induction treatment is recommended for at least as long as tumor shrinkage continues, or disease stabilization is maintained with tolerable toxicities [5]. In the TRIBE trial, 76% of patients received secondary treatment, of which 63% were on irinotecan-containing regimens [4]. In our BeTRI study, a sequential treatment following FOLFOXIRI plus bevacizumab was not intentionally defined as maintenance treatment with 5-fluorouracil and leucovorin plus bevacizumab. A total of 27 (59%) patients completed twelve cycles of protocol therapy. In fact, at the twelfth cycle, 18 (39.1%) patients received FOLFOXIRI plus bevacizumab, six (13.0%) patients received FOLFIRI plus bevacizumab, two (4.3%) patients received FOLFIRI, and one (2.2%) patient received infusional 5-fluorouracil and leucovorin plus bevacizumab. It should be noted that a FOLFIRI regimen was opted for eight (30.8%) patients. Moreover, R0 resection was performed in 10 (22.7%) patients including five patients treated with more than 12 cycles. These may have impacted on the encouraging lengths of 15.5 months for PFS and 37.1 months for OS. Taking the above into account, FOLFIRI appears to be suitable as a sequential treatment after three to four months of FOLFOXIRI or sooner for unacceptable neurotoxicity.

The current study had several potential limitations. A major weakness was that our trial was a single-arm, small sample size study with no comparators. Second, data on *BRAF* mutational status were not available. Finally, the follow-up time was short for evaluation of OS. In the future, appropriate sequential treatment strategies following FOLFOXIRI induction treatment should be considered.

In conclusion, our BeTRI study demonstrated that GONO-FOLFOXIRI plus bevacizumab were highly beneficial and manageable with toxicities in selected Japanese patients with good performance status who can tolerate intensive treatment.

## Electronic supplementary material

Below is the link to the electronic supplementary material.

Supplementary file1 (DOCX 24 kb)

Supplementary file2 (PPTX 228 kb)
