# Supplementary material for: A phase II study of FOLFOXIRI plus bevacizumab as initial chemotherapy for patients with untreated metastatic colorectal cancer: TRICC1414 (BeTRI)
Source: Int J Clin Oncol. 2020 Oct 23;26(2):399–408. doi: 10.1007/s10147-020-01811-w (PMC7819906; doi:10.1007/s10147-020-01811-w)
Supplement: Supplementary file 1 — Supplementary file1 (DOCX 24 kb) [file 10147_2020_1811_MOESM1_ESM.docx]

**Supplementary Table 1. Reduction level and dosage**

|  | Initial dosage | Level -1 | Level -2 | Level -3 |
| --- | --- | --- | --- | --- |
| L-OHP (mg/m^2^) | 80 | 65 | 50 | - |
| CPT-11 (mg/m^2^) | 165 | 150 | 125 | 100 |
| 5-FU (mg/m^2^) | 3200 | 2800 | 2400 | 2000 |
| *l*-LV (mg/m^2^) | 200 (fixed) | | | |
| Bevacizumab (mg/kg) | 5 (fixed) | | | |

Abbreviations: *L-OHP* oxaliplatin; *CPT-11* irinotecan; *5-FU* 5-fluorouracil: *l*-LV levofolinate

**Supplementary Table 2. Dose modifications for toxicities attributable to chemotherapy**

| PREVIOUS TOXICITY  (After resolution) | GRADE^*^ | L-OHP | CPT-11 | 5-FU |
| --- | --- | --- | --- | --- |
| Neutropenia with infection | 3 | -In principle, the dose should be reduced by one drug and one level at a time.  -Reduce the drug with higher dose level to -1 level.  -Reduce CPT-11, L-OHP, and 5-FU in that order, if the levels are the same. | | |
| Neutropenia with diarrhea | 3 |  |  |  |
| Neutropenia | 4 |  |  |  |
| Febrile Neutropenia | 4 |  |  |  |
| Diarrhea with fever (≥38℃) | Any |  |  |  |
| Thrombocytopenia | 3 |  |  |  |
| Thrombocytopenia  on scheduled treatment date | 2 |  |  |  |
| Others | 3 |  |  |  |
| Diarrhea | 3 |  | -Reduce the drug with higher dose level to -1 level. | |
| Mucositis oral | 3 |  |  | -Reduce to -1 level. |
| Palmar-plantar erythrodysesthesia syndrome | 3 |  |  |  |
| Peripheral neuropathy  on scheduled treatment date | 2 | -Reduce to -1 level.  Or  -Skip |  |  |
|  | 3 | -Stop |  |  |

Abbreviations: *L-OHP* oxaliplatin; *CPT-11* irinotecan; *5-FU* 5-fluorouracil

^*^Toxicities should be evaluated according to CTCAEv4.0.

Footnote: Even in the absence of toxicity that conflicts with the dose reduction criteria, the dose may be reduced if an investigator determines that a reduction is necessary to ensure the safety of the subject. In principle, the dose should be reduced by one drug and one level at a time, but an investigator may reduce the dose by two or three drugs at the same time or by two levels at his or her discretion. Once a dose has been reduced, it should not be increased later.

**Supplementary Table 3. Classification by primary tumor sidedness and *RAS* mutational status (FAS)**

|  | Right-sided colon  n (%) | Left-sided colon  n (%) | Total  n (%) |
| --- | --- | --- | --- |
| Wild *RAS* | 6 (13.6) | 9 (20.5) | 15 (34.1) |
| Mutated *RAS* | 3 (6.8) | 21 (47.7) | 24 (54.5) |
| unknown | 1 (2.3) | 4 (9.1) | 5 (11.4) |
| total | 10 (22.7) | 34 (77.3) | 44 (100) |

Abbreviations: *FAS* Full analysis set; *Right-sided colon* cecum, ascending and transverse colon; *Left-sided colon* descending, sigmoid colon, and rectum

**Supplementary Table 4. Second-line therapy (N=16)**

| Second-line treatment | Number of cases |
| --- | --- |
| FOLFIRI + anti-VEGF/VEGFR | 4 |
| FOLFOXIRI ± Bmab | 3 |
| Anti-EGFR ± CPT-11 | 3 |
| Capecitabine ± Bmab | 3 |
| CPT-11 + S-1 | 1 |
| Regorafenib | 1 |
| Trifluridine/tipiracil | 1 |

Abbreviations: *anti-VEGF/VEFR* bevacizumab (n=1), ramucirumab (n=2), zib-aflibercept (n=1); *Bmab* bevacizumab; *anti-EGFR* cetuximab (n=1), panitumumab (n=2); *CPT-11* irinotecan; *S-1* tegafur/gimeracil/oteracil
