# Supplementary material for: A phase II study of FOLFOXIRI plus bevacizumab as initial chemotherapy for patients with untreated metastatic colorectal cancer: TRICC1414 (BeTRI)
Source: Int J Clin Oncol. 2020 Oct 23;26(2):399–408. doi: 10.1007/s10147-020-01811-w (PMC7819906; doi:10.1007/s10147-020-01811-w)
Supplement: Supplementary file 2 — Supplementary file2 (PPTX 228 kb) [file 10147_2020_1811_MOESM2_ESM.pptx]

## Slide 1
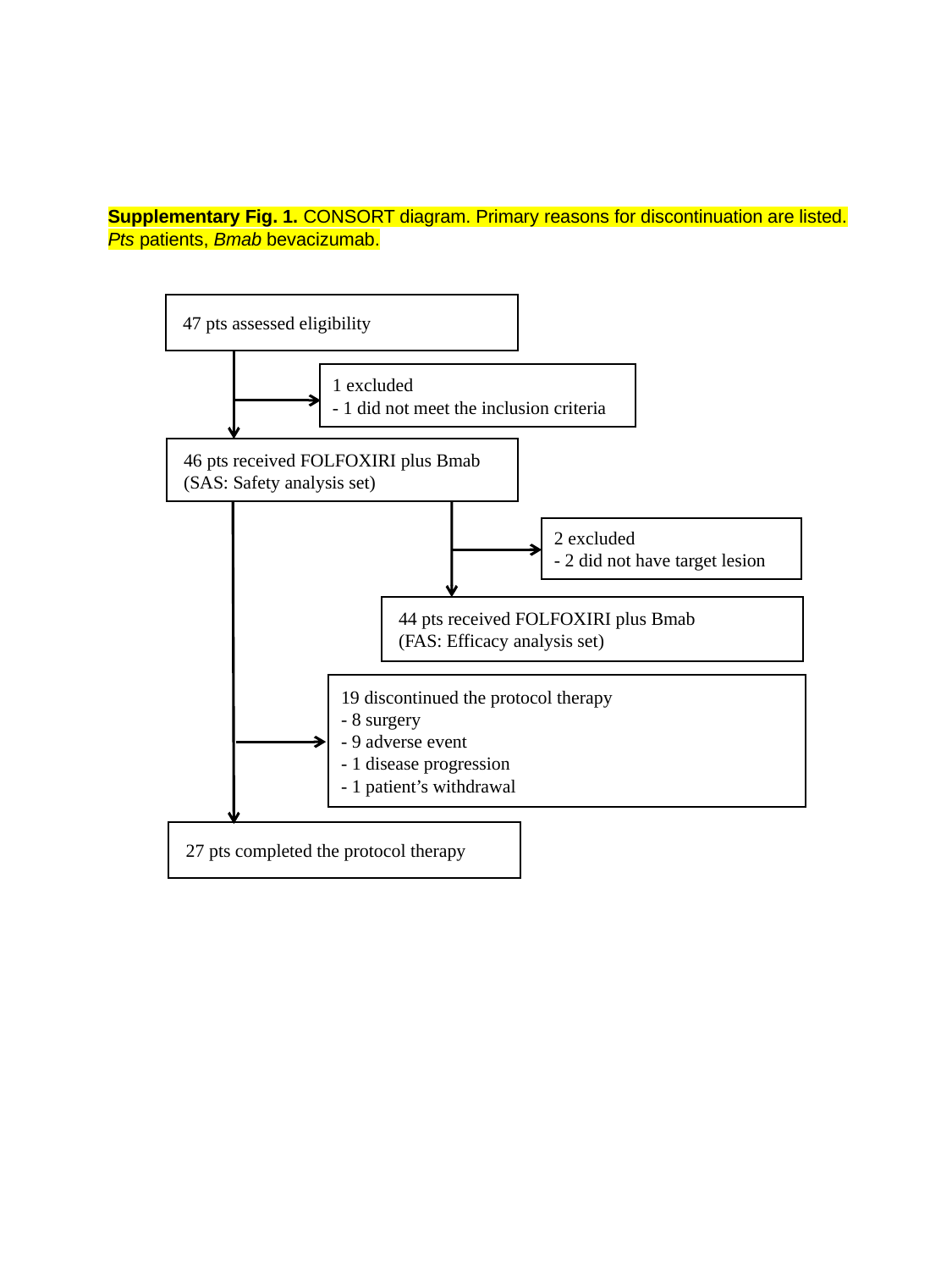

Supplementary Fig. 1. CONSORT diagram. Primary reasons for discontinuation are listed. Pts patients, Bmab bevacizumab.
 47 pts assessed eligibility
1 excluded
- 1 did not meet the inclusion criteria
 46 pts received FOLFOXIRI plus Bmab
 (SAS: Safety analysis set)
2 excluded
- 2 did not have target lesion
 44 pts received FOLFOXIRI plus Bmab
 (FAS: Efficacy analysis set)
19 discontinued the protocol therapy
- 8 surgery
- 9 adverse event
- 1 disease progression
- 1 patient’s withdrawal
 27 pts completed the protocol therapy

## Slide 2
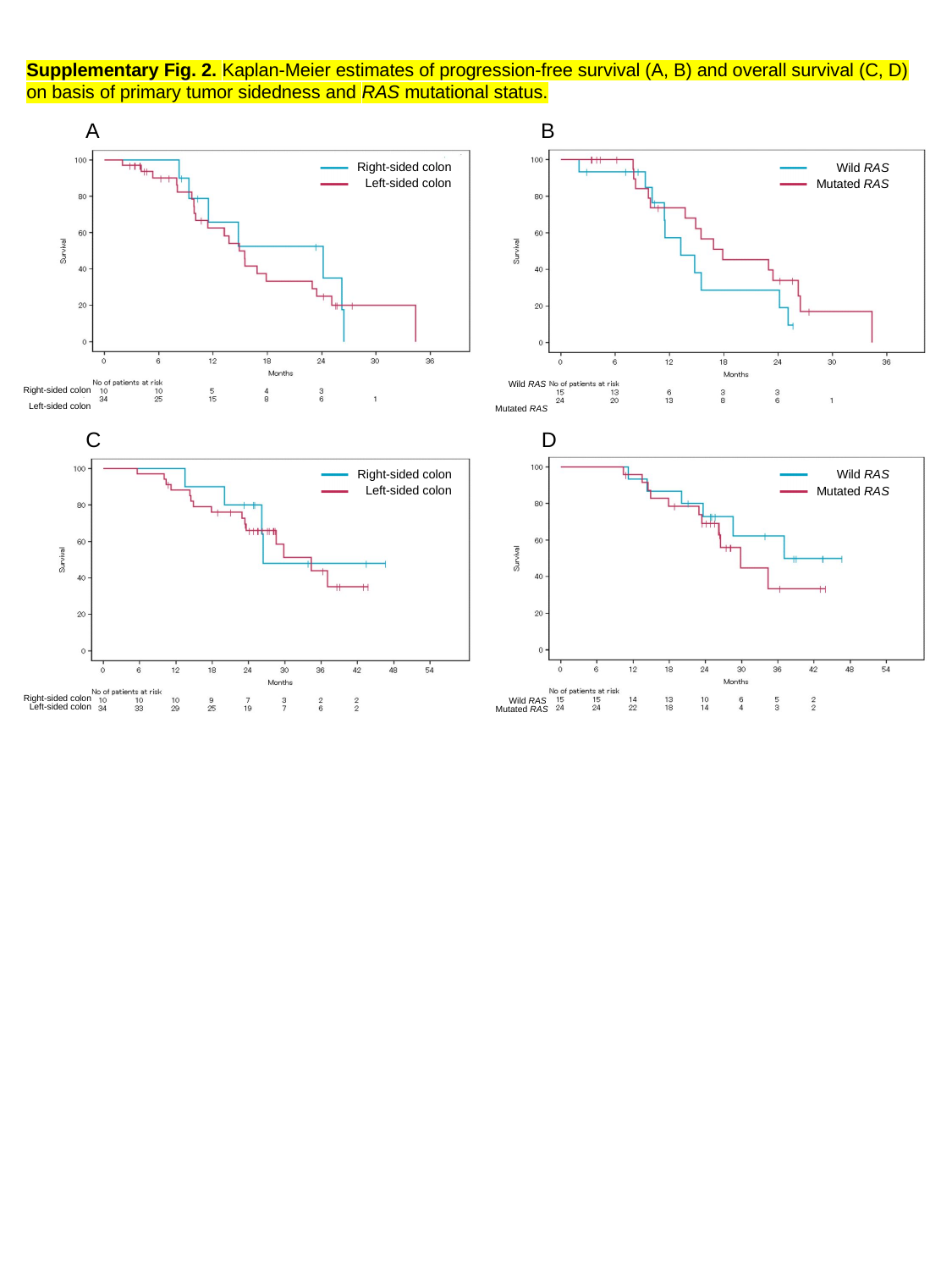

Supplementary Fig. 2. Kaplan-Meier estimates of progression-free survival (A, B) and overall survival (C, D) on basis of primary tumor sidedness and RAS mutational status.
A
B
Right-sided colon
Wild RAS
Left-sided colon
Mutated RAS
Wild RAS
Right-sided colon
Left-sided colon
Mutated RAS
D
C
Right-sided colon
Wild RAS
Left-sided colon
Mutated RAS
Right-sided colon
Wild RAS
Left-sided colon
Mutated RAS
